# Analysis of risk factors for capsular contracture after breast augmentation: a retrospective study

**DOI:** 10.3389/fsurg.2025.1596993

**Published:** 2025-05-20

**Authors:** Xin-Yao Li, Shi-Qi Xiao

**Affiliations:** ^1^ Department of Plastic Surgery, Shenzhen Yixing Medical Beauty Hospital, Shenzhen, Guangdong, China; ^2^ School of Food and Drug, Shenzhen Polytechnic University, Shenzhen, Guangdong, China

**Keywords:** capsular contracture, breast augmentation, smoking, oversized implants, hematoma

## Abstract

**Background:**

Capsular contracture is a prevalent complication following breast augmentation that adversely affects aesthetic outcomes and often necessitates revision surgery. This study aimed to assess potential predictors of capsular contracture in patients undergoing primary breast augmentation.

**Methods:**

In this retrospective study, 212 patients (386 implant insertions) who underwent primary breast augmentation with silicone implants at our institution between January 2019 and December 2022 were included. Patients were stratified based on the presence of capsular contracture, diagnosed and graded by board-certified plastic surgeons using the Baker classification system. Demographic, clinical, and surgical parameters—including age, body mass index (BMI), smoking history, lactation history, implant characteristics, surgical approach, and postoperative drainage volume—were recorded. Statistical analyses were performed using SPSS version 26.0 (Statistical Package for the Social Sciences). Univariate analysis was conducted with *t*-tests and chi-square tests, while multivariate logistic regression was applied to identify independent risk factors, with significance set at *P* < 0.05.

**Results:**

Univariate analysis demonstrated that variables such as age, BMI, and drainage volume did not significantly differ between groups. However, smoking history, oversized implants, and hematoma occurrence were significantly associated with capsular contracture. Multivariate logistic regression confirmed that a positive smoking history [odds ratio [OR] = 2.95, 95% confidence interval [CI]: 1.38–6.38], implant oversizing (OR = 6.00, 95% CI: 2.44–14.80), and hematoma formation (OR = 14.60, 95% CI: 1.78–118.90) independently increased the risk.

**Conclusions:**

This study identifies smoking, implant oversizing, and hematoma as significant modifiable risk factors for capsular contracture following breast augmentation. These findings underscore the importance of careful preoperative planning and patient counseling to mitigate postoperative complications.

## 1 Introduction

Capsular contracture remains one of the most significant complications associated with breast augmentation surgery, adversely impacting patient satisfaction and potentially necessitating revision procedures. Defined as a pathological fibrotic reaction leading to tightening and deformation of the fibrous capsule surrounding a breast implant, capsular contracture can result in aesthetic dissatisfaction, discomfort, and diminished quality of life (1, 2). Despite advances in surgical techniques and implant technologies, the precise etiology and contributing risk factors for capsular contracture remain multifactorial and incompletely understood. Identifying these risk factors is critical for developing preventive strategies and enhancing patient outcomes.

Previous studies have implicated several patient-specific and procedural factors in the development of capsular contracture (3, 4). Patient-related factors include age, smoking status, body mass index (BMI), and genetic predispositions influencing inflammatory responses and wound healing. Procedural factors encompass implant characteristics such as type (saline or silicone), surface texture (smooth or textured), placement plane (subglandular vs. submuscular), and surgical technique employed, including aseptic measures and postoperative management protocols. Among these, implant surface texture and placement plane have attracted considerable research attention, with studies reporting variable outcomes regarding their impact on the incidence and severity of capsular contracture. Implant surface texturing was initially developed to minimize capsular contracture by disrupting the linear alignment of collagen fibers during capsule formation. However, recent clinical investigations have yielded conflicting results, with some evidence suggesting textured implants might reduce contracture rates (5), while others report similar or increased risks compared to smooth implants (6). The discrepancy in findings underscores the complexity of host-tissue reactions and the potential influence of additional variables such as microbial contamination, surgical technique, and postoperative management (7–9).

This retrospective study aims to systematically analyze the risk factors associated with capsular contracture following breast augmentation surgery by examining a comprehensive set of demographic, procedural, and implant-related variables. Through detailed statistical analysis, this research seeks to provide robust evidence clarifying the relative impact of identified factors, thereby aiding clinicians in risk stratification, patient counseling, and development of targeted preventive strategies.

## 2 Methods

### 2.1 Study design

This retrospective study evaluated patients who underwent breast augmentation surgery at our institution between January 2019 and December 2022. A total of 212 patients, accounting for 386 implant insertions, were included in the study and stratified into two groups based on the occurrence of capsular contracture: the capsular contracture group (*n* = 31) and the control group (*n* = 355). Informed consent was obtained from all participants. The study's methodology and protocols were reviewed and approved by our hospital's ethics committee. All procedures complied with relevant guidelines and the ethical principles of the Declaration of Helsinki. Data was handled confidentially, with personal identifiers removed to protect participant privacy.

### 2.2 Inclusion and exclusion criteria

The inclusion criteria for this study encompassed all patients who underwent primary breast augmentation with silicone implants at our institution between January 2019 and December 2022, provided that complete clinical and surgical records were available and that a minimum postoperative follow-up duration of 12 months was achieved. Patients who met these criteria and consented to the use of their anonymized data for research purposes were included in the analysis. Conversely, the exclusion criteria comprised patients with a prior history of breast surgery, those undergoing concurrent reconstructive procedures, and individuals with documented systemic or autoimmune disorders known to affect wound healing. Additionally, patients with incomplete medical records or insufficient follow-up data were excluded from the study.

### 2.3 Assessment of capsular contracture severity

The diagnosis and grading of capsular contracture were performed by board-certified plastic surgeons using the Baker classification system (10). In this study, postoperative capsular contracture was categorized into four distinct grades: Grade I was characterized by a soft breast texture with a natural appearance; Grade II exhibited a firm breast consistency while maintaining an overall natural contour; Grade III was defined by significant firmness accompanied by minor implant deformation; and Grade IV was distinguished by pronounced firmness with severe implant deformation, often associated with pain or tenderness.

### 2.4 Surgical technique and postoperative follow-up protocol

Preoperative measurements, including breast base width, nipple-to-inframammary fold distance, and soft tissue thickness, were obtained for all patients using the measurement method described by Tebbetts et al. Implant selection, encompassing both type and size, was determined based on the High Five preoperative design system in conjunction with patient preferences (11). The surgical approach was tailored to patient wishes, with incisions made via either the axillary or inframammary route. In all cases, implants were placed in a subpectoral plane, specifically beneath the pectoralis major muscle. Following the establishment of the implant pocket, the surgical site was irrigated using physiological saline solution supplemented with a combination of cephalosporin and gentamicin, as previously described (7). A negative pressure drainage tube was routinely placed prior to the insertion of the implant, and the incision was closed in standard fashion. Postoperatively, drainage tubes were removed when the 24-h unilateral drainage volume did not exceed 30 ml and the drainage fluid maintained a stable color without evidence of deepening. Patients were scheduled for follow-up evaluations at 1, 3-, 6-, 12-, and 24-months post-surgery, during which the presence and severity of capsular contracture were systematically assessed.

### 2.5 Data collection and outcome measures

The study recorded demographic and clinical parameters for both patient cohorts, including age, body mass index (BMI), smoking history, lactation history, surgical approach, implant brand, implant type and surface characteristics, implant oversizing, utilization of endoscopy, drainage volume, and the occurrence of hematoma. In our institution, the average implant size is approximately 350 ml, which corresponds to typical anatomical dimensions among our patient population. Implant oversizing was defined as an implant volume exceeding the recommended capacity according to the High Five preoperative design system by 20% or more.

### 2.6 Statistical analysis

Statistical analyses were executed using SPSS version 26.0. Quantitative data conforming to a normal distribution were expressed as mean ± standard deviation, whereas non-normally distributed variables were presented as median with interquartile range (Q1, Q3). Intergroup comparisons for continuous variables were performed utilizing the *t*-test, while categorical data, expressed as counts and/or percentages, were analyzed using the chi-square test. Initially, univariate analyses were conducted to investigate the relationship between the recorded parameters and the incidence of capsular contracture following breast augmentation. Subsequently, multivariate logistic regression was employed to ascertain the independent risk factors associated with the development of capsular contracture. All statistical tests were two-tailed, and a *p*-value of less than 0.05 was considered indicative of statistical significance.

## 3 Results

### 3.1 Patient cohort and capsular contracture severity

A total of 232 breast augmentation procedures were performed on female patients at our institution. Thirteen patients were lost to follow-up and seven cases had incomplete medical records, resulting in a final cohort of 212 patients with ages ranging from 20 to 61 years (mean age, 31.3 years). Utilizing a per-breast analysis, 386 implant insertions were evaluated and subsequently stratified into two groups based on the occurrence of capsular contracture: a capsular contracture group (*n* = 31) and a control group (*n* = 355). Among the 212 consecutively included patients, 174 (82.1%) underwent bilateral primary breast augmentation, whereas 38 (17.9%) underwent unilateral primary augmentation, resulting in a total of 386 implant insertions. Unilateral augmentation was performed either to correct pronounced pre-existing breast asymmetry (*n* = 28) or to address congenital unilateral hypoplasia, such as in cases of Poland sequence or tuberous breast deformity (*n* = 10). Within the capsular contracture cohort, the severity of contracture was graded according to the Baker classification, with 21 cases classified as Baker Grade II, 3 as Baker Grade III, and 7 as Baker Grade IV.

### 3.2 Univariate analysis of variables associated with capsular contracture

Univariate analysis was performed to compare clinical and surgical variables between patients with capsular contracture and controls following breast augmentation (Table 1). No statistically significant differences were observed in age (30.2 ± 6.1 vs. 32.1 ± 7.2 years; *t* = 1.43, *P* = 0.16), body mass index (18.9 ± 1.9 vs. 18.8 ± 1.8 kg·m^−2^; *t* = 0.30, *P* = 0.77), or drainage volume (70.3 ± 42.1 vs. 64.6 ± 36.8 ml; *t* = 0.82, *P* = 0.41) between the two groups. Similarly, lactation history, implant surface properties, implant brand, surgical approach, and the use of endoscopy did not differ significantly (all *P* > 0.05). Conversely, significant associations were identified for several variables. The capsular contracture group exhibited a markedly higher proportion of patients with a positive smoking history (64.5% vs. 31.0%; *χ*^2^ = 14.43, *P* < 0.01) compared to the control group. Moreover, the incidence of oversized prosthesis was significantly elevated among patients with capsular contracture, with 25.8% classified as having an implant volume exceeding the recommended threshold, as opposed to only 6.9% in the control group (*χ*^2^ = 13.61, *P* < 0.01). In addition, the occurrence of hematoma was significantly more common in the capsular contracture cohort (6.5% vs. 0.6%; *χ*^2^ = 9.64, *P* = 0.024).

**Table 1:** Univariate analysis of capsular contracture following breast augmentation.

Variable	Capsular contracture group (*n* = 31)	Control group (*n* = 355)	*χ*^2^/t value	*P* value
Age (years)	30.2 ± 6.1	32.1 ± 7.2	1.43	0.16
BMI (kg·m^−2^)	18.9 ± 1.9	18.8 ± 1.8	0.30	0.77
Drainage volume (ml)	70.3 ± 42.1	64.6 ± 36.8	0.82	0.41
Lactation history			0.68	0.41
No	11 (35.5%)	101 (28.5%)		
Yes	20 (64.5%)	254 (71.5%)		
Implant surface properties			0.01	0.91
Textured	21 (67.7%)	244 (68.7%)		
Smooth	10 (32.3%)	111 (31.3%)		
Implant brand			1.8	0.75
Mentor	21 (67.7%)	229 (64.5%)		
Allura	8 (25.8%)	78 (22%)		
Polytech	2 (6.5%)	37 (10.4%)		
McGonagall	0 (0.0%)	11 (3.1%)		
Surgical approach			0.45	0.50
Underarm	29 (93.5%)	341 (96.1%)		
Inferior fold	2 (6.5%)	14 (3.9%)		
Endoscope use			1.34	0.25
No	8 (25.8%)	62 (17.5%)		
Yes	23 (74.2%)	293 (82.5%)		
Smoking history			14.43	<0.01
No	11 (35.5%)	245 (69.0%)		
Yes	20 (64.5%)	110 (31.0%)		
Oversized prosthesis			13.61	<0.01
No	23 (74.2%)	331 (93.2%)		
Yes	8 (25.8%)	24 (6.8%)		
Hematoma			9.64	0.024
No	29 (93.5%)	353 (99.4%)		
Yes	2 (6.5%)	2 (0.6%)		

BMI, body mass index.

### 3.3 Multivariate analysis of risk factors associated with capsular contracture

Multivariate logistic regression analysis was conducted to identify independent predictors of capsular contracture, adjusting for potential confounding variables including surgical approach, implant surface properties, and the use of endoscopic assistance. The analysis revealed that a history of smoking was significantly associated with an increased risk of capsular contracture (OR = 2.95, 95% CI: 1.38–6.38, *P* < 0.05). Additionally, patients receiving oversized implants—defined as an implant volume exceeding the recommended capacity by 20%—demonstrated a markedly higher likelihood of developing capsular contracture (OR = 6.00, 95% CI: 2.44–14.80, *P* < 0.05). Hematoma formation was also identified as a significant risk factor, with affected patients exhibiting a substantially elevated risk (OR = 14.60, 95% CI: 1.78–118.90, *P* < 0.05) (Table 2).

**Table 2:** Multivariate logistic regression analysis of capsular contracture following breast augmentation.

Variable	Level	OR (95% CI)	*P* value
Implant surface properties	Textured (reference)	1	–
	Smooth	0.95 (0.43–2.07)	0.89
Surgical approach	Underarm (reference)	1	–
	Inferior fold	2.32 (0.61–8.74)	0.22
Endoscope used	No (reference)	1	–
	Yes	0.85 (0.36–2.05)	0.23
Oversized prosthesis	No (reference)	1	–
	Yes	6.00 (2.44–14.80)	<0.01
Smoking history	No (reference)	1	–
	Yes	2.95 (1.38–6.38)	<0.01
Hematoma	No (reference)	1	–
	Yes	14.60 (1.78–118.90)	<0.01

### 3.4 *Post-hoc* power analysis

A comprehensive *post-hoc* power evaluation was undertaken for all eleven candidate covariates (smoking history, oversized implant, hematoma, age, implant brand, endoscope use, surgical approach, lactation history, 24-h drainage volume, BMI, and implant surface). A weighted *post-hoc* power analysis was performed to evaluate the overall analytical quality of the study, by integrating the relative importance of all 11 candidate predictors. The weights for each variable were calculated based on their individual univariate test statistics (*χ*^2^ for categorical variables, squared *t*-values for continuous variables), thereby reflecting each variable's statistical contribution. This approach balances statistical impact with clinical relevance and helps to mitigate the influence of outliers or extreme values. The weighted overall power was calculated to be 0.81 (81%), indicating that, at the current sample size, the study retains a reasonably strong capacity to detect the combined effect of these predictors.

## 4 Discussion

Breast augmentation is among the most frequently performed cosmetic surgical procedures worldwide, offering significant improvements in body contour and patient self-esteem. Despite advances in surgical techniques and implant technology, capsular contracture remains a persistent and vexing complication. This condition, characterized by the excessive fibrotic encapsulation of the implant, often results in pain, distortion of breast shape, and the necessity for revision surgery, thereby negatively impacting patient satisfaction and clinical outcomes (5, 12, 13). This retrospective study aimed to identify risk factors associated with capsular contracture following breast augmentation. Both univariate and multivariate analyses were performed to evaluate potential clinical and surgical predictors. Our findings indicate that while demographic factors such as age, body mass index (BMI), drainage volume, lactation history, implant surface properties, implant brand, surgical approach, and the use of endoscopic assistance were not significantly associated with capsular contracture, several other factors were. Notably, a positive smoking history, the use of oversized implants, and the occurrence of hematoma emerged as significant independent predictors of capsular contracture.

The multivariate logistic regression analysis demonstrated that patients with a history of smoking exhibited nearly a threefold increased risk of developing capsular contracture compared to nonsmokers (OR = 2.95, 95% CI: 1.38–6.38, *P* < 0.05). Smoking is well recognized to impair wound healing by inducing vasoconstriction and reducing tissue oxygenation, which may lead to chronic inflammation. This chronic inflammatory state likely results in an exaggerated fibroblastic response and increased collagen deposition around the implant, thereby predisposing to capsular contracture. Furthermore, smoking has been associated with alterations in immune function, which could exacerbate the local inflammatory response following surgical intervention (14, 15). These mechanistic insights are supported by previous studies indicating that smoking adversely affects postoperative outcomes in a variety of surgical procedures.

Oversized implants, defined in our study as those with volumes exceeding the recommended capacity by 20%, were found to significantly increase the risk of capsular contracture (OR = 6.00, 95% CI: 2.44–14.80, *P* < 0.05). The use of implants larger than the physiologically appropriate size may impose excessive tension on the surrounding tissues. This tension can result in a heightened inflammatory response and subsequent fibrotic encapsulation as the tissues attempt to adapt to the mechanical stress. The phenomenon of tissue overdistension may also compromise local microcirculation, further augmenting the inflammatory cascade and predisposing to fibrosis. The strong association observed in our study suggests that adherence to size recommendations is crucial in mitigating the risk of capsular contracture (9, 16, 17). Hematoma formation was another significant predictor identified, with affected patients demonstrating an approximately 14-fold increased risk of developing capsular contracture (OR = 14.60, 95% CI: 1.78–118.90, *P* < 0.05). Hematoma may serve as a nidus for inflammation and subsequent fibrosis by providing a substrate for the infiltration of inflammatory cells and promoting the release of pro-fibrotic cytokines. The presence of a hematoma can disrupt normal tissue architecture and prolong the inflammatory phase of wound healing, thereby facilitating the formation of a dense, fibrous capsule. Moreover, the resorption of hematoma is often associated with the deposition of hemosiderin and other byproducts that can further stimulate fibrotic pathways (18, 19).

Interestingly, our analysis did not find significant associations between capsular contracture and other variables such as age, BMI, drainage volume, lactation history, implant surface properties, implant brand, surgical approach, or the use of endoscopy. The lack of association with age and BMI suggests that patient-specific metabolic or systemic factors may be less influential in the pathogenesis of capsular contracture compared to local factors at the implant-tissue interface. Although implant surface properties and brand have been the focus of considerable investigation, our findings corroborate previous reports indicating that these factors may not independently predict the development of capsular contracture when other risk factors are considered. Similarly, the surgical approach and the utilization of endoscopic assistance did not significantly influence the risk, indicating that, within the context of standardized surgical techniques, these variables might be less critical determinants of capsular contracture (3, 20).

The implications of these findings are clinically significant. Surgeons should be cognizant of the potential deleterious effects of smoking on postoperative outcomes and consider implementing preoperative smoking cessation protocols. Moreover, careful preoperative planning with regard to implant size selection is paramount. Adherence to established guidelines for implant volume may mitigate excessive tissue stress and reduce the subsequent risk of fibrotic capsule formation. Additionally, meticulous intraoperative hemostasis and postoperative management to prevent hematoma formation are essential strategies for minimizing the risk of capsular contracture.

These recent studies, when considered alongside our findings, offer a more comprehensive perspective on the multifactorial nature of capsular contracture risk across both reconstructive and aesthetic contexts. Moon et al. (21) demonstrated that subpectoral implant placement and direct-to-implant reconstruction significantly increased the incidence and severity of capsular contracture in irradiated patients. In contrast, our study focused on primary aesthetic augmentations without confounding from radiotherapy, thereby isolating modifiable surgical risk factors. This strengthens the generalizability of our findings to non-oncologic populations. Munhoz et al. (22) analyzed complication risks in secondary hybrid augmentations and found capsular contracture to be common across both fat-grafted and non-grafted groups. While their cohort involved reoperative procedures with prior pocket alterations, our study exclusively evaluated primary augmentations, minimizing surgical history bias and allowing clearer attribution of risk to smoking, hematoma, and oversizing. Sánchez-Delgado et al. (23) identified trauma and subglandular placement as significant risk factors in a general implant population, while other variables lacked statistical significance. Our multivariate model builds upon this by confirming independent associations for hematoma, implant oversizing, and smoking, supported by *post-hoc* power analysis, thus offering stronger inferential validity.

This study has several limitations that warrant consideration. The retrospective design may have introduced selection bias, and the relatively small size of the capsular contracture group represents a major limitation of this study and introduces a high risk of bias, particularly in subgroup analyses. Additionally, although adjustments were made for several potential confounders, unmeasured factors might have influenced the observed associations. The study was conducted at a single institution, which may restrict the generalizability of the findings to broader populations with varying surgical techniques and patient demographics. Furthermore, while the focus was on select clinical and procedural variables, other potentially relevant factors—such as genetic predisposition or detailed immunologic profiles—were not evaluated. Future research should aim to address these limitations by employing prospective, multicenter designs with larger patient cohorts. Moreover, incorporating advanced imaging modalities and biomarker analyses could further elucidate the underlying mechanisms of capsular contracture and foster the development of more effective prevention and treatment strategies.

## 5 Conclusions

In conclusion, this retrospective study identified smoking, implant oversizing, and hematoma as significant risk factors for capsular contracture following breast augmentation. These modifiable factors highlight the need for careful preoperative planning and patient counseling to improve surgical outcomes. Future prospective studies may further elucidate underlying mechanisms, thereby facilitating more effective prevention and management strategies.

## Data Availability

The raw data supporting the conclusions of this article will be made available by the authors, without undue reservation.
